# The chronic pain coping inventory: Confirmatory factor analysis of the French version

**DOI:** 10.1186/1471-2474-7-13

**Published:** 2006-02-14

**Authors:** Manon Truchon, Denis Côté, Sandrine Irachabal

**Affiliations:** 1 Centre for interdisciplinary research in rehabilitation and social integration, 525, boul. Hamel, Québec, QC, G1M 2S8, Canada; 2 Département des relations industrielles, Université Laval, Québec, QC, G1K 7P4, Canada; 3 Université Pierre Mendès France Grenoble II, IUT II, département carrières sociales, 2 place Doyen Gosse, 38031 Grenoble cedex, France; 4 Equipe de psychologie de la santé, Laboratoire EA526, Université Victor Segalen Bordeaux II, 3ter Place de la Victoire, 33000 Bordeaux, France

## Abstract

**Background:**

Coping strategies are among the psychosocial factors hypothesized to contribute to the development of chronic musculoskeletal disability. The Chronic Pain Coping Inventory (CPCI) was developed to assess eight behavioral coping strategies targeted in multidisciplinary pain treatment (Guarding, Resting, Asking for Assistance, Task Persistence, Relaxation, Exercise/Stretch, Coping Self-Statements and Seeking Social Support). The present study had two objectives. First, it aimed at measuring the internal consistency and the construct validity of the French version of the CPCI. Second, it aimed to verify if, as suggested by the CPCI authors, the scales of this instrument can be grouped according to the following coping families: Illness-focused coping and Wellness-focused coping.

**Method:**

The CPCI was translated into French with the forward and backward translation procedure. To evaluate internal consistency, Cronbach's alphas were computed. Construct validity of the inventory was estimated through confirmatory factor analysis (CFA) in two samples: a group of 439 Quebecois workers on sick leave in the sub-acute stage of low back pain (less than 84 days after the work accident) and a group of 388 French chronic pain patients seen in a pain clinic. A CFA was also performed to evaluate if the CPCI scales were grouped into two coping families (i.e. Wellness-focused and Illness-focused coping).

**Results:**

The French version of the CPCI had adequate internal consistency in both samples. The CFA confirmed the eight-scale structure of the CPCI. A series of second-order CFA confirmed the composition of the Illness-focused family of coping (Guarding, Resting and Asking for Assistance). However, the composition of the Wellness-focused family of coping (Relaxation, Exercise/Stretch, Coping Self-Statements and Seeking Social Support) was different than the one proposed by the authors of the CPCI. Also, a positive correlation was observed between Illness and Wellness coping families.

**Conclusion:**

The present study indicates that the internal consistency and construct validity of the French version of the CPCI were adequate, but the grouping and labeling of the CPCI families of coping are debatable and deserve further analysis in the context of musculoskeletal and pain rehabilitation.

## Background

In 1991, Jensen et al. [1] published an exhaustive review of the literature on the beliefs and adaptation strategies used by individuals suffering from chronic pain as well as on the measuring instruments most frequently used in research on these individuals. Following this review, they developed a new pain-related coping measuring instrument: the Chronic Pain Coping Inventory (CPCI) [2]. The CPCI mainly aims to measure the behavioral coping strategies frequently targeted in the context of multidisciplinary programs for chronic pain management. They are strategies that are either encouraged (e.g., exercise) or discouraged (e.g., rest) in these intervention programs.

Jensen et al. [2] explained the CPCI development steps and presented the results of the first two studies that made it possible to document certain psychometric qualities of the instrument: internal consistency, test-retest reliability, criterion validity and agreement with a significant-other version. This initial validation process resulted in the proposal of a version of the CPCI consisting of 64 items measuring eight ways of coping (Guarding, Resting, Asking for Assistance, Relaxation, Task Persistence, Exercise/Stretch, Coping Self-Statement, and Seeking Social Support). Jensen et al. [2] suggested that the CPCI ways of coping were grouped into two families according to whether they are wellness-focused or illness-focused, except for Seeking Social Support which was considered as belonging to another unidentified family. The results of two studies carried out by Jensen et al. [2] indicated that internal consistency and test-retest reliability of the proposed scales were adequate. To document criterion validity, Jensen et al. [2] calculated correlation coefficients between the CPCI scales and measures of functioning (e.g., depression, pain-related distress, functional status). The results indicated that four scales are more directly associated with functioning: Guarding, Resting, Asking for Assistance and Task Persistence.

The psychometric qualities of the CPCI proved to be sufficiently satisfactory to encourage further efforts to validate this tool. Until now, five other studies carried out on different versions of the CPCI demonstrated its validity and reliability [3-7]. However, only two studies have analyzed the factorial structure of the instrument. Hadjistavropoulos et al. [3] analyzed the factorial structure of the CPCI using exploratory factor analysis (principal component analysis). This analysis reproduced, with a few differences, the eight factors suggested by Jensen et al. [2]. It should be noted that the scales were initially developed conceptually and statistically only through the examination of item-scale correlation. Hadjistavropoulos et al. encouraged future validation work on the CPCI using confirmatory factor analysis (CFA).

Recently, Tan et al. [4] used CFA to examine the factorial structure of the CPCI. Although they reproduced the proposed factorial structure, a few limitations hindered their conclusions. Firstly, the CFA lacked power with a ratio of subjects to free parameters of less than 4:1 whereas it was suggested to perform CFA with a minimum ratio of 10:1 [8]. Nevertheless, this criticism is lessened by the quality of the psychometric qualities of the CPCI reported in previous studies. It should be noted that the size of the CPCI (64 items) makes it very difficult to recruit a sufficient sample (N > 1500). Secondly, no fit indices were reported in Tan et al. paper which was contrary to usual practice [9]. Considering the permanent controversy concerning the evaluation of model fit, it was impossible to evaluate the fit of the model independently. Finally, although they apparently reported all the pattern coefficients over 0,30, no structure coefficients were reported. Structure coefficients are as important as pattern coefficients in the interpretation of CFA (or any analysis under the general linear model) [10,11].

The present study has two objectives. The first consisted of analyzing internal consistency as well as the construct validity of the French version of the CPCI in continuity with Tan et al. study [4]. The second objective consisted of verifying whether, as originally proposed by Jensen et al. [2], the CPCI ways of coping were grouped according to the Illness and Wellness families of coping.

## Methods

### Participants

#### Québec sample

Participants were workers on sick leave compensated by the Quebec Occupational health and safety commission (Commission de la santé et de la sécurité du travail, CSST) after a work accident involving the low back region. They participated in a larger study of biopsychosocial determinants of chronic disability due to non-specific low back pain. The following inclusion criteria were used: age between 18 and 60, a first or new compensated episode of low back pain in the last 12 months, on sick leave between 3 and 12 weeks after the accident (i.e., in the sub-acute stage). The following exclusion criteria were used: insufficient understanding of French, returned to work, pregnancy, previous back surgery, and severe spinal pathology such as fracture, tumor, infection, cauda equina syndrome, and symptoms suggesting nerve compression.

The CSST provided 4374 potential subjects during a 16-month period. Among the 3326 contacted, 2132 were excluded, 379 refused to participate and 815 persons initially accepted to participate (initial acceptance rate = 815/(815 + 379 + 1048) = 36%. After initial acceptance, 439 workers participated to the initial assessment, 51 were returned to work and 325 refused (participation rate = 439/(439 + 325) = 57%. Main reasons for non-participation were that workers were already returned to work (1727), 693 did not met inclusion/exclusion criteria, and 457 refused to participate. The workers were between 18 and 60 years of age (M = 38 years, SD = 10.2) and more than half were men (59%)). Most were married or part of a couple (61.3%) and they earned between Can $10,000 and $30,000 (59.7%) (mean income of Quebecois workers in 2002 was near Can $ 29,000). The majority had a high school diploma (62%) or higher (23%). More information for this sample is available in Truchon and Côté [12].

This project was approved by the Ethics Committee of the Institut en réadaptation en déficience physique de Québec (march 25^th ^2002).

#### French sample

The 388 subjects making up this sample were recruited in the context of the "Unité de Traitement des Douleurs Chroniques" of the "Centre Hospitalier Universitaire de Bordeaux". The purpose of this unit consisting of neurologists, psychiatrists, psychologists, physiatrists, kinesiologists, rheumatologists and acupuncturists is to treat chronic pain in the context of overall and multidisciplinary management. The subjects included in this study were mainly women (70%) from 18 to 70 years of age (M = 44, SD = 12.6), suffering from headache (59%), back pain (21%) or neuropathic pain (20%) for at least 6 months (M = 15 years, SD = 13.7) and not suffering from major neurological and/psychiatric impairment.

### Measure

#### The Chronic Pain Coping Inventory

The CPCI is a self-report measure asking persons to rate the frequency of use of behavioral and cognitive coping strategies. The strategies are grouped into the following 8 subscales: Guarding, Resting, Asking for Assistance, Relaxation, Task Persistence, Exercise/Stretch, Seeking Social Support, and Coping Self-Statements. The Likert response scale contained 8 levels in the original version. The French version contained 4 levels: never (0), sometimes (1), often (2) and very often (3). All the items in a scale had to be completed in order to calculate a mean score. Otherwise, the scale score was considered as missing. Mean scale scores vary from 0 to 3.

Translation from English to French was done by an English-born translator. This preliminary French version was translated back into English by a French-born translator. The two English versions were judged equivalent by a third translator and the French version was deemed adequate. A few items are slightly different to represent linguistic differences between France and Québec [see Additional file 1].

### Procedure

#### Québec sample

Once the eligible workers agreed to participate, a booklet containing all the questionnaires was sent by mail to their homes. Some workers were instructed to complete the booklet and send it back in the pre-stamped envelope. They were contacted regularly if they had not sent back their booklet. Another group of workers who were visited by a nurse were asked to complete the booklet before the nurse's visit. All the booklets were checked on reception and the workers were called back to complete missing information that was considered crucial.

#### French sample

Part of the sample (145 subjects) was met with on several occasions in their management by a psychologist who, during one meeting, asked the patients to complete different questionnaires including the CPCI. These 145 individuals participated in a semi-prospective study on the cognitive and behavioral reactions of chronic pain subjects. The second part of the sample completed the questionnaire after having given consent at the end of a consultation with one of the practitioners in the unit.

### Analysis

All statistical analyses were done with SPPS (Statistical Package for the Social Sciences, version 11.0) except for confirmatory factor analyses which were executed in EQS (Structural Equation Modeling Software, versions 5.7 b and 6.1).

The factorial structure of the CPCI items was examined with a first-order CFA. Eight factors were constructed to test the adequacy of the scales. Even though the samples were fairly large (≈400), the high number of items (62) made it imprudent to perform a powerful single CFA including all the items. Based on the recommended ratio of number of subjects to the number of model parameters (152), both samples were far from this 10:1 ratio [8]. Therefore, the samples were combined to form a sample of 827 subjects to obtain a better ratio (5.4:1) but still short of the recommendation. The power of the analysis will be addressed in the Discussion.

The hierarchical factorial structure of the CPCI scales was examined with a second-order CFA in which a second level of factors was set to speak for first-order factors. Contrarily to the first-order analysis, the second-order CFA were run separately in each samples. In order to respect a 10:1 ratio of subjects to free parameters, the scale scores could have been used as measured variables. But to obtain better estimates of ways of coping, the items were grouped into 16 parcels. Such grouping produced around 40 parameters to be estimated depending on the model tested and therefore respected a ratio of subjects to free parameters of 10 to 1. Two parcels per scale were constituted from the original file. Therefore, the number of items constituting a parcel varied for each scale. Each pair of parcels was examined and modified to obtain equivalent parcels [13]. The parcel data are available on request.

The following rules were used to evaluate model fit: χ^2^/df ratio smaller than 3; CFI larger than .90, SRMR smaller than or equal to .08 and RMSEA smaller than .08 or ideally smaller than .05. The coefficients were also taken into account in the evaluation of models considering they should be high and similar in magnitude.

## Results

### Missing data

The French sample was complete. The Canadian sample had less than 1% missing responses. Descriptive statistics and correlations were computed with complete cases only. A single imputation of the missing responses was performed with the Expectation-Maximization algorithm of SPSS for each scale separately. This complete file was used with the first-order CFA where items served as indicators. For the second-order CFA, parcels were computed with completes cases which resulted in 2.2% missing parcels. No imputation of parcels was performed but the EQS maximum likelihood procedure for missing data was used.

### Descriptive statistics

Table 1 presents means, standard deviations and internal consistency coefficients of the CPCI scales in both samples. The pattern of means was slightly different across samples. The internal consistency indices were all satisfactory (> .70) with the exception of the Relaxation scale in the French sample (α = .68). Globally, these results reflect the adequate construction of the scales by Jensen et al. [2].

Table 2 presents correlations between the CPCI scales in both samples. The correlation matrix revealed that most of the scales were positively correlated, except for Task Persistence, which was negatively correlated with the Illness-focused scales (Guarding, Resting, and Asking for Assistance). Individual item-scale correlations are presented in Table 3. Except for items 40 and 50, the item-scale correlations were adequate, ranging from .33 to .81. Based on their poor item-scale correlation, items 40 and 50 were removed from all the analyses, including those presented in Table 1 and 2.

### First-order construct validity (ways of coping)

For identification purposes, the variance of the eight factors was set to 1.00. Because ordinal data might not conform to the normality assumption required by the maximum likelihood estimation, the robust Satorra-Bentler scaled χ^2 ^was reported as well as its derived indices (the χ^2 ^to degrees of freedom ratio, the Comparative Fit Index [CFI], the root mean square error approximation [RMSEA]). The residuals were also examined individually as well as summarized by the standardized root mean square residual (SRMR).

Coefficients and squared correlations for each item are presented in Table 3. Whereas some fit indices were satisfactory (χ^2^_SB _= 5529.9 (1801), χ^2^_SB_/df = 3.1, RMSEA_SB _(90% CI) = .050 (.049 – .052), SRMR = .07), the CFI_SB _(.82) was low and 11% of the standardized residuals were over |0.1|. It should be noted that factorial structures with more than five indicators by factor are difficult to confirm, and the CFI and the RMSEA are affected by the number of items [14]. Nevertheless, the majority of the coefficients were larger than .60 and all the items had their highest coefficients with their parent scale. This indicated conceptual homogeneity among the items within each scale and a certain level of heterogeneity between the scales. There were few problematic items: item 39 ("held part of my body (e.g. arm) in a special position) had the lowest coefficient (.28) and four items of the Coping Self-Statements scale had a coefficient lower than .50 (items 14, 21, 27, 37).

### Second-order construct validity (families of coping)

With the Québec sample, a series of CFA were conducted to evaluate Jensen's hypothesis about the structure of the coping scales. In the models, the eight CPCI ways of coping were first-order factors, and a second-order level represented families of coping. For statistical identification purposes, each first-order factor had one path set to 1.00, and the variance of second-order factors was set at 1.00. CFA were run with the parcel score as raw data, and the covariance matrix was analyzed.

The first-order level was considered adequate with pattern coefficients ranging between .60 and .99. Examination of first-order structure coefficients revealed that each pair of parcels always had the highest coefficients with their respective first-order factor, and there were no equivocal parcels.

The first second-order model tested (model A) included only one factor predicting the eight CPCI latent ways of coping (see Table 5). This model was inadequate based on the fit indices as well as on the pattern coefficients; the factor was not homogeneous. Model B was a test of Jensen's hypothesis of an Illness family (F1; Guarding, Resting, Asking) and a Wellness family (F2; Relaxation, Task Persistence, Exercise/Stretch, Coping Self-Statements); Seeking Social Support was let free to covary with both second-order factors. This model was a significant improvement over model A according to the fit indices. Three elements were evident in model B. First, the size of pattern coefficients of F1 showed a homogeneous factor. Second, Task Persistence was not related to F2 (Wellness) as proposed by Jensen. Third, Seeking Social Support was moderately related to both factors. In model C, Task Persistence was switched to F1. Model C was a slight improvement over model B. Interestingly, Task Persistence correlated negatively with F1, suggesting that it is an inverted measure of F1. The multivariate Lagrange Multiplier test indicated a significant omitted relationship between Task Persistence and Coping Self-Statements. To account for this omitted relationship without complicating the model, model D had the Task Persistence latent scale predicted both by F1 and F2. Model D was a slight improvement over all the preceding models. Both pattern coefficients of Task Persistence were significant but moderate (-.62 and .47). Because dual paths should be avoided in measurement models, model E was tested in which Task Persistence was allowed to covary with both factors. Also, based on the size of the coefficients, Seeking Social Support was set to be predicted by F2. Although model E was slightly worse than model D, it was better than models A to C. In general, model E had satisfactory fit indices. F1 was homogeneous and constituted an asset of the model. Task Persistence correlated moderately only with F1. The only deficiency of model E was the range of pattern coefficients of F2 (.49 to .91), indicating a moderate lack of homogeneity among this family of coping. Structure coefficients of the final model are also presented in Table 5 and show that ways of coping are mainly associated with their parent family.

Models B and E were also tested in the French sample. Results presented in Table 5 show a very similar pattern, and only model E provides an adequate fit.

## Discussion

The present study had two objectives. First, it aimed to document the internal consistency as well as construct validity of the French version of the CPCI of Jensen et al. [2]. Second, it aimed to verify whether, as proposed by Jensen et al., the CPCI ways of coping are grouped according to two families of coping: Illness-focused coping and Wellness-focused coping.

Regarding internal consistency, the Cronbach's alpha coefficients obtained in the two samples were adequate. The coefficients ranged from .75 to .91 in the Québec sample, and from .75 to .92 in the French sample, except for the Relaxation scale for which the internal consistency coefficient was .68 in the French sample. In the Québec sample, the Relaxation scale was also the one with the lowest alpha coefficient (i.e., .74). Jensen [15], in an article on the validation of questionnaires, stated that coefficients of .90 or more demonstrate an excellent internal consistency, while coefficients between .70 and .90 suggested that internal consistency was adequate. With coefficients below .70, internal consistency cannot be said to be adequate. Hence, studies employing the CPCI should systematically compute internal consistency coefficients and pay attention to the Relaxation scale. Overall, the coefficients obtained in the two samples of the present study were comparable to those obtained by Jensen et al. [2] with the original version of the CPCI as well as to those obtained by Ektor-Andersen et al. [7] with the Swedish version. In the study of Ektor-Andersen et al., the Relaxation scale had a coefficient of .72, which was also the lowest of the inventory.

Regarding the poor quality of eliminated items 40 (Rested in a chair or recliner) and 50 (Used self-hypnosis to relax), this indicated the necessity of reviewing the formulation of these items or eliminating them from the French version of the CPCI. We propose that item 50 could be eliminated because self-hypnosis was a very rarely employed strategy in our samples; it has the lowest mean of the 64 items in both samples. As for item 40, it seemed that it did not fit any factor measured by the CPCI.

The first-order confirmatory factorial analysis indicated that the CPCI scales were homogeneous conceptually. This reflected the meticulous construction of the scales by Jensen et al. [2]. Construct validity was also shown by Hadjistavropoulos et al. [3]. Through exploratory factorial analysis, they were able to find the structure proposed by Jensen et al. with a few differences. It should be noted that Jensen et al. [2] did not use factorial analysis to specify the eight factors of the CPCI, but used the item-total correlations. The present study confirmed the quality of the individual scales and the overall factorial structure of the CPCI. Our results, in combination with those obtained by Jensen et al. [2], Hadjistavropoulos et al. [3] and more recently by Tan et al. [4], supported the eight-factor structure of the CPCI, but this time for the French version of the instrument. It was reassuring to observe the same factorial structure despite the fact that our sample and Tan's were under the recommended size to perform CFA [8].

We strongly urge the use of shorter versions of the CPCI for clinical and research purposes. Jensen's group already provided versions with 42, 16 and 8 items [5,16]. This work should be confirmed and continued using best practices for reducing questionnaires' length [17].

Second-order confirmatory factorial analysis was used to verify the grouping of scales of the CPCI into two major coping families. According to Jensen et al. [2], an initial family would consist of strategies (Task Persistence, Relaxation, Exercise/Stretch and Coping Self-Statements) said to be focused on well-being and encouraged during treatments (wellness-focused coping). A second family would consist of strategies (Guarding, Resting and Asking for Assistance) focused on disease and discouraged during treatments (Illness-focused coping). The Seeking Social Support strategy, which is neither encouraged nor discouraged during treatment, was thought to belong to another unidentified family. Five possible groupings of coping scales were tested on the Québec sample. A model with a single factor (model A) was tested in order to obtain a basis for comparison with dual models. This model was inadequate. The results obtained with the four dual models (B, C, D, E) indicated overall that the first factor (F1) was stable and homogeneous. In other words, the Illness family proposed by Jensen et al. [2], comprising the Guarding, Resting and Asking for Assistance ways of coping, was confirmed. On the other hand, the confirmation of the Wellness family was not achieved based on statistical and conceptual grounds. Statistically, the fit of models B to E was acceptable and improving. But Task Persistence had to be tested in different positions in order to find its membership. In fact, Task Persistence found its place outside the factors tested, although it was moderately related to F1 as revealed by its structure coefficients (-.41) in model E. The negative relationship seems to suggest that Task Persistence is an inverted measure of F1, although somewhat weak. In all the models tested including final model E, the F2 family suffers from a moderate lack of homogeneity because the coefficients were not as high and equivalent as in the F1 family despite the fact that the fits were acceptable. Besides the exclusion of Task Persistence of the F2 family, the other change from Jensen's proposal was the inclusion of Seeking Social Support in the F2 family, although the results in the French sample do not firmly support this inclusion.

On conceptual grounds, it seems inappropriate to label F2 a Wellness family because F1 and F2 were positively correlated and because a parallel study of the predictive validity of the CPCI in the Canadian sample has shown that the scales composing the F2 family were unrelated or actually positively related with disability, pain, and depressive mood as were the F1 ways of coping [12].

It should be noted that the two groupings (Wellness and Illness) were meant to reflect associations of the scales with functional measures (disability, pain, and mood) and not associations among the scales. In this respect, this study only addressed the hierarchical organization of pain-related coping strategies. It is a possibility that Wellness and Illness families could be positively correlated while related to different outcomes. A person can simultaneously apply contradictory coping strategies. However, previous studies of the CPCI as well as our own companion study cast doubts of the adaptive nature of coping comprising the F2 family.

Overall, the observed results indicated that internal consistency of the version translated into French of the CPCI was adequate. Also, they indicated that this version has good construct validity. It may therefore be used in future studies. However, a series of confirmatory factorial analyses indicated that the grouping of the CPCI ways of coping into an Illness family and a Wellness family is debatable. In fact, only the Illness factor could be adequately reproduced within the Québec and French samples. According to a recently proposed classification of coping, this family describes the avoidance or escape from a non-contingent environment [18]. In line with this classification, we propose that this CPCI family of coping should be called the Avoidance family to more accurately define its nature. Consequently, the relationship of this coping family with fear-avoidance beliefs and distress should be explored in the context of chronic pain development [19,20]. Similarly, the Wellness label should be dropped because it does not reflect Jensen's original proposal and because it is not necessarily related to a favorable rehabilitation [12]. Unless this discrepancy is resolved, the exact nature and label of the F2 family remains uncertain.

## Conclusion

To our knowledge, the present study is the first to verify whether, as suggested by Jensen et al. [2], the scales making up the CPCI are grouped into families of coping with the use of confirmatory factorial analysis. It would therefore be desirable that future studies use this type of analysis to reveal the hierarchical nature of the coping processes. Such an approach would make it possible to assess the functional homogeneity and distinctiveness of the coping families as well as their adaptive value in pain rehabilitation [18].

## Competing interests

The author(s) declare that they have no competing interests.

## Authors' contributions

SI led the translation process and collected the data in France. MT developed the Canadian study in which the inventory was used and wrote the article. DC performed the statistical analyses and helped with writing the article. All three of the authors read and approved the article.

## Pre-publication history

The pre-publication history for this paper can be accessed here:



## Figures and Tables

**Table 1:** Mean, standard deviation, internal consistency of the CPCI scales

	Canadian sample			French sample		
		
Scale	Mean	Sd	α (95% CI)	Mean	Sd	α (95% CI)
Guarding	1.6	.57	.77 – **.80 **– .82	1.1	.67	.79 – **.81 **– .84
Resting^†^	1.7	.56	.78 – **.81 **– .84	1.6	.76	.83 – **.85 **– .88
Asking	1.5	.73	.79 – **.82 **– .84	1.0	.72	.71 – **.75 **– .79
Relaxation^‡^	0.8	.49	.72 – **.75 **– .79	0.8	.55	.63 – **.68 **– .73
Task p.	0.8	.54	.76 – **.79 **– .82	1.3	.63	.73 – **.76 **– .80
Exercise	1.1	.64	.86 – **.88 **– .90	0.5	.62	.90 – **.92 **– .93
Coping S.-S.	1.3	.60	.86 – **.88 **– .89	1.3	.62	.84 – **.86 **– .88
S. Social S.	0.9	.65	.90 – **.91 **– .92	0.8	.64	.86 – **.88 **– .90

† item 40 removed. ‡ item 50 removed.

**Table 2:** Correlations between CPCI scales in the Canadian and French samples

Scale	1	2	3	4	5	6	7	8
1. Guarding	-	.44*	.55*	.28*	-.19*	.22*	.11*	.14*
2. Resting†	.59*	-	.42*	.22*	-.37*	.05	.05	.08
3. Asking	.56*	.53*	-	.15*	-.23*	.07	.03	.28*
4. Relaxation‡	.30*	.37*	.26*	-	.03	.57*	.42*	.31*
5. Task P.	-.25*	-.25*	-.29*	.02	-	.07	.27*	.03
6. Exercise	.12*	.12*	.04	.39*	.13*	-	.30*	.22*
7. Coping S.-S.	.24*	.21*	.15*	.47*	.23*	.33*	-	.36*
8. S. Social S.	.29*	.39*	.41*	.41*	.00	.17*	.37*	-

Note. Canadian sample in the lower matrix, French sample in the upper matrix. Pairwise deletion was used in the Canadian sample. The French sample was complete.

* p < .05. † item 40 removed. ‡ item 50 removed.

**Table 3:** Item-scale correlations and coefficients of the first-order CFA

item	item-scale (Canada)	item-scale (France)	gua.	res.	ask.	rela.	task	exe.	css	sss	r^2^
11	.50	.50	**.53**	.33	.39	.21	-.25	.16	.08	.12	.28
15	.48	.49	**.60**	.37	.43	.23	-.28	.18	.09	.14	.35
33	.56	.59	**.71**	.44	.51	.27	-.33	.22	.10	.17	.50
35	.44	.50	**.52**	.33	.38	.20	-.25	.16	.08	.12	.27
39	.38	.35	**.28**	.18	.21	.11	-.13	.09	.04	.07	.08
41	.53	.63	**.61**	.38	.45	.24	-.29	.19	.09	.14	.38
46	.59	.62	**.74**	.46	.54	.29	-.35	.23	.11	.17	.55
48	.53	.60	**.74**	.46	.54	.29	-.35	.23	.11	.17	.55
56	.42	.35	**.55**	.34	.40	.21	-.26	.17	.08	.13	.30
05	.55	.63	.45	**.72**	.40	.27	-.36	.11	.07	.18	.52
07	.59	.55	.41	**.65**	.36	.25	-.33	.10	.07	.17	.42
18	.63	.70	.49	**.79**	.44	.30	-.40	.12	.08	.20	.62
40	.19	.24									
47	.65	.71	.48	**.77**	.43	.29	-.39	.12	.08	.20	.60
58	.53	.66	.40	**.64**	.36	.24	-.32	.10	.07	.16	.41
64	.40	.54	.31	**.50**	.28	.19	-.25	.08	.05	.13	.25
09	.69	.57	.53	.41	**.73**	.25	-.32	.17	.07	.29	.53
25	.76	.66	.61	.47	**.84**	.28	-.37	.20	.08	.34	.70
42	.64	.58	.58	.45	**.80**	.27	-.36	.19	.08	.32	.64
62	.47	.38	.34	.26	**.47**	.16	-.21	.11	.04	.19	.22
01	.51	.38	.19	.19	.17	**.50**	-.04	.30	.26	.24	.25
12	.46	.50	.25	.24	.22	**.64**	-.05	.39	.34	.30	.41
24	.45	.43	.22	.22	.19	**.57**	-.04	.34	.30	.27	.32
31	.49	.37	.19	.19	.17	**.50**	-.04	.30	.26	.24	.25
36	.53	.40	.19	.19	.17	**.50**	-.04	.30	.26	.23	.25
50	.18	.25									
59	.49	.45	.22	.22	.19	**.57**	-.04	.35	.30	.27	.32
02	.52	.56	-.34	-.36	-.32	-.05	**.72**	-.07	.17	-.04	.52
04	.55	.40	-.25	-.27	-.24	-.04	**.53**	-.05	.12	-.03	.28
28	.51	.50	-.28	-.29	-.26	-.04	**.58**	-.06	.13	-.03	.34
34	.58	.51	-.29	-.31	-.28	-.05	**.62**	-.06	.14	-.04	.38
51	.61	.54	-.36	-.38	-.34	-.06	**.75**	-.08	.17	-.04	.57
63	.50	.52	-.27	-.29	-.25	-.04	**.57**	-.06	.13	-.03	.32
03	.52	.62	.20	.10	.16	.41	-.07	**.67**	.19	.15	.45
13	.49	.59	.18	.09	.14	.36	-.06	**.60**	.17	.13	.36
17	.58	.70	.20	.10	.16	.41	-.07	**.67**	.19	.15	.45
26	.43	.60	.17	.09	.13	.34	-.06	**.56**	.16	.13	.32
29	.68	.74	.24	.12	.18	.47	-.08	**.77**	.22	.17	.60
32	.33	.42	.11	.06	.09	.22	-.04	**.36**	.10	.08	.13
38	.56	.68	.22	.11	.17	.43	-.07	**.71**	.20	.16	.51
43	.59	.55	.20	.10	.15	.39	-.07	**.64**	.18	.14	.41
52	.59	.65	.21	.10	.16	.41	-.07	**.68**	.19	.15	.46
55	.67	.79	.24	.12	.19	.49	-.08	**.80**	.23	.18	.64
60	.73	.83	.26	.13	.20	.51	-.09	**.84**	.24	.19	.71
61	.69	.78	.24	.12	.18	.47	-.08	**.77**	.22	.17	.60
10	.51	.62	.09	.06	.06	.32	.14	.17	**.61**	.25	.37
14	.50	.40	.06	.04	.04	.21	.09	.11	**.40**	.17	.16
19	.74	.65	.11	.08	.07	.41	.18	.22	**.78**	.32	.61
21	.46	.44	.07	.05	.04	.24	.11	.13	**.46**	.19	.21
23	.63	.58	.09	.07	.06	.33	.15	.18	**.63**	.26	.40
27	.46	.43	.06	.04	.04	.22	.10	.12	**.42**	.17	.18
30	.57	.51	.09	.07	.06	.33	.14	.18	**.63**	.26	.39
37	.46	.45	.07	.05	.04	.24	.11	.13	**.46**	.19	.21
45	.75	.75	.12	.09	.08	.43	.19	.23	**.82**	.34	.68
49	.68	.55	.10	.07	.06	.36	.16	.19	**.67**	.28	.45
54	.65	.71	.11	.08	.07	.39	.17	.21	**.74**	.30	.55
06	.67	.61	.16	.17	.27	.32	-.04	.15	.28	**.68**	.46
08	.78	.71	.18	.20	.31	.37	-.05	.17	.32	**.78**	.61
16	.64	.58	.15	.17	.27	.31	-.04	.15	.27	**.66**	.44
20	.69	.66	.17	.19	.29	.34	-.04	.16	.30	**.73**	.53
22	.72	.52	.16	.17	.27	.32	-.04	.15	.28	**.67**	.45
44	.81	.71	.19	.21	.33	.38	-.05	.18	.33	**.81**	.65
53	.65	.66	.16	.18	.28	.33	-.04	.16	.29	**.70**	.49
57	.74	.72	.18	.20	.31	.36	-.05	.17	.32	**.77**	.59

Pattern/structure coefficients of free parameters are in bold. All other pattern coefficients fixed to zero are not shown. Structure coefficients (in normal text) represent the correlation between an item and the factors.

**Table 4:** Second-order confirmatory factor analyses: fit indices, standardized pattern coefficients and structure coefficients

	Model (Canadian sample)									Model (French sample)			
	
	A	B		C		D		E		B		E	
**Factors**	F1	F1	F2	F1	F2	F1	F2	F1	F2	F1	F2	F1	F2

**Ways of coping**													
Guarding	**.82**	**.86**		**.86**		**.85**		**.86**	*.46*	**.90**		**.84**	*.28*
Resting	**.86**	**.88**		**.89**		**.88**		**.89**	*.48*	**.62**		**.69**	*.23*
Asking	**.77**	**.79**		**.79**		**.79**		**.78**	*.42*	**.77**		**.74**	*.25*
Task P.	**-.30**		**.07^a^**	**-.37**		**-.62**	**.47**	*-.41*	*.13*		**.07^a^**	*-.46*	*.11*
Relaxation	**.62**		**.97**		**.99**		**.90**	*.49*	**.91**		**.96**	*.32*	**.96**
Exercise	**.23**		**.52**		**.51**		**.52**	*.27*	**.49**		**.71**	*.23*	**.69**
CSS	**.37**		**.65**		**.63**		**.70**	*.36*	**.67**		**.54**	*.19*	**.56**
SSS	**.56**	*.48*	*.59*	*.47*	*.59*	*.48*	*.61*	*.34*	**.64**	*.23*	*.44*	*.15*	**.45**
F1		*-*	*.47*	*-*	*.46*	*-*	*.47*	*-*	*.54*		*.32*		*.34*
**Fit indices**													
χ^2^_SB_	389.0	247.3		211.5		176.6		192.0		249.5		236.0	
Df	96	94		94		93		94		94		94	
χ^2^_SB_/df	4.1	2.6		2.3		1.9		2.0		2.7		2.5	
SRMR	.11	.09		.08		.06		.07		.10		.08	
CFI_SB_	.91	.96		.97		.98		.97		.95		.95	
RMSEA_SB_	.08 (.08–.09)	.06 (.05–.07)		.05 (.04–.06)		.05 (.04–.06)		.05 (.04–.06)		.07 (.06–.08)		.06 (.05–.07)	
% residuals larger than |0.1|	26	12		12		9		12		21		17	

a. All pattern coefficients (in bold) are significant (p < .05), except where indicated. Structure coefficients are presented in italics.
